# Phospholipids of the Plasma Membrane – Regulators or Consequence of Cell Polarity?

**DOI:** 10.3389/fcell.2020.00277

**Published:** 2020-04-28

**Authors:** Michael P. Krahn

**Affiliations:** Department of Medical Cell Biology, Medical Clinic D, University Hospital of Münster, Münster, Germany

**Keywords:** cell polarity, phospholipids, phosphoinositide, phosphatidic acid, plasma membrane

## Abstract

Cell polarity is a key feature of many eukaryotic cells, including neurons, epithelia, endothelia and asymmetrically dividing stem cells. Apart from the specific localization of proteins to distinct domains of the plasma membrane, most of these cells exhibit an asymmetric distribution of phospholipids within the plasma membrane too. Notably, research over the last years has revealed that many known conserved regulators of apical-basal polarity in epithelial cells are capable of binding to phospholipids, which in turn regulate the localization and to some extent the function of these proteins. Conversely, phospholipid-modifying enzymes are recruited and controlled by polarity regulators, demonstrating an elaborated balance between asymmetrically localized proteins and phospholipids, which are enriched in certain (micro)domains of the plasma membrane. In this review, we will focus on our current understanding of apical-basal polarity and the implication of phospholipids within the plasma membrane during the cell polarization of epithelia and migrating cells.

## Introduction

One of the fundamental prerequisites for the function of epithelial cells is the polarization along their apical-basal axis. This apical-basal polarity is established and regulated by a set of highly conserved polarity determinants (Table 1), which act in antagonizing fashions in order to keep the balance between the apical and the basolateral plasma membrane domain. Notably, most key determinants of this epithelial apical-basal polarity also regulate anterior-posterior polarity in the zygote of *Caenorhabditis elegans* and the oocyte of *Drosophila* as well as front-rear polarity in migrating cells (for review see St Johnston and Ahringer, 2010; Tepass, 2012; Rodriguez-Boulan and Macara, 2014; Campanale et al., 2017).

**TABLE 1:** Summary of conserved polarity regulators and their reported phospholipid-binding capacity.

**Mammalian**	***Drosophila***	***C. elegans***	**Phospholipid-binding**
**Apical**			
PAR-3	Bazooka	PAR-3	PI(4,5)P2, PI(3,4,5)P3
PAR-6	PAR-6	PAR-6	–
PKCζ/ι	aPKC	PKC-3	–
Cdc42	Cdc42	Cdc42	PS
Rac1	Rac1	Rac-2	PA, PI(4,5)P2, PI(3,4,5)P3
Tiam1	–	–	PI(3,4)P2, PI(4,5)P2, PI(3,4,5)P3
PTEN	PTEN	DAF-18	PI(4,5)P2, (PS, PC)
Annexin-2	–	–	PI(4,5)P2
Crb1/2/3	Crb	Crb-1	–
Pals1	Stardust	Magu-2	–
PATJ	PATJ	Mpz-1	–
**Basolateral**			
Llgl	Lgl	Lgl-1	PI(4)P, PI(4,5)P2
Dlg1	Dlg	Dlg-1	–
Scrb	Scrb	Let-413	–
MARK3	PAR-1	PAR-1	PS, PA, PI(4)P
LKB1	LKB1	PAR-4	PA

**FIGURE 1:**
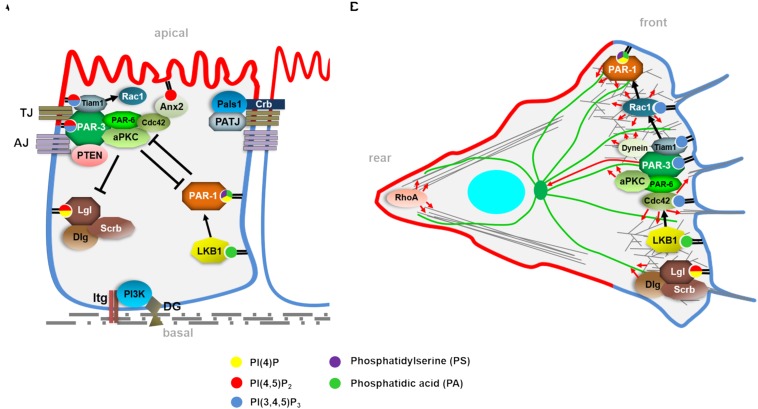
**(A)** Simplified scheme of a differentiated epithelial cell with apical-basal polarity regulators and their described phospholipid interaction. Apical-basal polarization starts with the recruitment and activation of PI3-Kinase to focal adhesions (Integrin-complexes) and to the Dystroglycan complex, determining the basal side of the epithelial cell. Subsequently, the PAR/aPKC-complex (PAR-3, PAR-6, aPKC) is localized to the Tight Junctions (TJ) by PAR-3. PAR-3 also recruits PTEN, which catalyzes the turn over from PI(3,4,5)P3 to PI(4,5)P2. Furthermore, PAR-3 binds the Rac1 activator Tiam1 and PAR-6/aPKC target Cdc42 to the complex. Together and partly in redundancy with the second TJ-associated complex, the Crb-complex (Crb, Pals1, and PATJ), the PAR/aPKC complex determines the PI(4,5)P2-enriched apical plasma membrane domain, which is counterbalanced by the basolateral cell polarity regulators. Here, the Lgl/Dlg/Scrb-module and the kinase LKB1/PAR-1 also exhibit phospholipid-binding capacities, which are essential for their localization and function. **(B)** Polarity complexes and phospholipids regulating cell migration. In contrast to apical-basal polarity, migrating cells exhibit a front-rear polarity with apical and basolateral polarity regulators localizing at the leading edge, regulating cell protrusions (lamellipodia in particular via Rac1 and filopodia via Cdc42) by modulating the actin cytoskeleton (gray fibers) or affecting microtubules (green fibers).

In order to achieve this balance, polarity determinants cluster in apical and basolateral polarity complexes: In particular, the PAR/aPKC-complex and the Crumbs complex determine the apical plasma membrane domain, whereas the Scribble (Scrb)/Discs Large (Dlg)/Lethal (2) Giant Larvae (Lgl) complex together with the kinases PAR-1 and LKB1 (PAR-4 in *C. elegans*) substantiate the (baso-) lateral domain (Figure 1). The core components of the PAR/aPKC complex are the scaffolding proteins PAR-3 (Bazooka in *Drosophila*) and PAR-6 (PAR-6α/β/γ in mammals) and the Serine/Threonine kinase aPKC (atypical protein kinase C, Protein Kinase Cζ and ι in mammals). Furthermore, PAR-6 is regulated by the small GTPase Cdc42, which dynamically associates with the PAR-complex too.

Within the Crumbs (Crb)-complex, the transmembrane protein Crb is stabilized in the plasma membrane by its adaptor protein Pals1 (Stardust in *Drosophila*), which in turn recruits the adaptor proteins Pals1-associated tight junction protein (PATJ) and Lin-7 to the complex (reviewed by Bulgakova and Knust, 2009). However, several studies demonstrated a crosstalk between these two apical complexes, indicating that their composition is highly dynamic and depends on the cell type, differentiation status and other stimuli of the epithelium (Hurd et al., 2003b; Gao and Macara, 2004; Lemmers et al., 2004; Penkert et al., 2004; Sotillos et al., 2004; Wang et al., 2004; Kempkens et al., 2006; Krahn et al., 2010a; Sen et al., 2015; Whitney et al., 2016). The basolateral localized Scrb, Dlg and Lgl are scaffolding proteins which function as a module to determine basolateral plasma membrane domain and to regulate the assembly of cell–cell contacts. Notably, deletion of these components results not only in polarity defects, but also in tissue overgrowth (in *Drosophila* and to some extent in vertebrates), leading to the identification of these proteins as tumor suppressors (reviewed by Stephens et al., 2018).

In order to mutually exclude apical and basolateral determinants, aPKC phosphorylates Lgl and PAR-1, which subsequently dissociate from the plasma membrane in the aPKC-active apical zone of epithelia and apical-basal polarized neural stem cells (neuroblasts) of *Drosophila* (Betschinger et al., 2003; Plant et al., 2003; Hurov et al., 2004; Suzuki et al., 2004; Wirtz-Peitz et al., 2008; Doerflinger et al., 2010). Conversely, PAR-1 phosphorylates PAR-3 and aPKC, displacing them from the basolateral cortex (Benton and St Johnston, 2003; Hurd et al., 2003a; Krahn et al., 2009). In *Drosophila* neuroblasts, aPKC also excludes the adaptor protein Miranda and the Notch inhibitor Numb from the basal cortex by phosphorylation, thereby controlling asymmetric cell division (Smith et al., 2007; Atwood and Prehoda, 2009).

**FIGURE 2:**
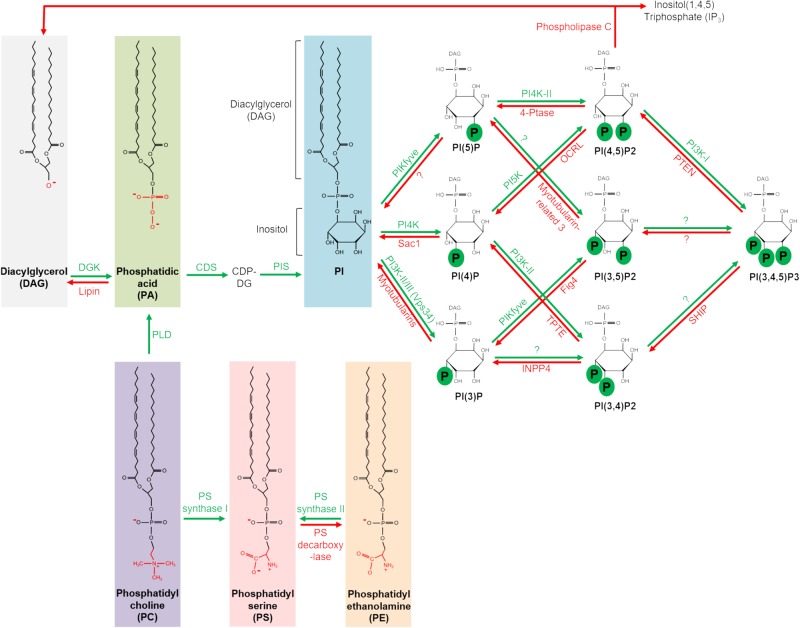
Metabolism of major phospholipids implicated in cell polarity. DGK, diacylglycerol kinase. CDP-DG, cytidine diphosphate diacylglycerol. CDS, CDP-diacylglycerol synthase. FIG4, FIG4 phosphoinositide 5-phosphatase. FYVE-type zinc finger containing. INPP4, inositol polyphosphate-4-phosphatase. OCRL, OCRL inositol polyphosphate 5-phosphatase. PIKfyve, phosphoinositide kinase. PIS, PI synthase. PTEN, phosphatase and tensin homolog. SHIP, Src homology 2 (SH2) domain containing inositol polyphosphate 5-phosphatase. TPTE, transmembrane phosphatase with tensin homology.

Phospholipids are a major component of biological membranes and not only responsible for dynamic membrane fluctuations but also function as signaling hubs (for review see Liu et al., 2013; Schink et al., 2016; Yang et al., 2018; Kay and Fairn, 2019). Phosphatidylcholine (PC), phosphatidylethanolamine (PE), phosphatidylserine (PS) and sphingomyelin are most frequent and constitute the framework of biological membranes, stabilized by cholesterol. However, the less abundant phosphatidic acid (PA) and phosphoinositides (PI) have been found to play crucial roles in recruiting membrane-associated proteins and function as signaling hubs. Moreover, the accumulation of distinct phospholipids (in particular of the PI family) is a characteristic feature of different cellular compartments, targeting phospholipid-binding proteins to these compartments. An overview of the generation and metabolism of the main phospholipids discussed in this review is given in Figure 2.

## Protein–Phospholipid Interactions

Several distinct lipid-binding domains have been identified in proteins (reviewed by Varnai et al., 2017): for instance, Pleckstrin homology (PH) domains and Epsin N-terminal homology (ENTH) domains bind preferentially to PI(4,5)P2 and PI(3,4,5)P3. FYVE domains target endosomal proteins to PI(3)P-enriched endosomes. C1 domains in PKCs bind to diacylglycerol, which activates the kinase and C2 domains recognize acidic phospholipids. However, over the last years, an increasing amount of proteins, which do not contain a distinct lipid-binding domain, have been described to directly associate with phospholipids. Mapping the interaction domains, positively charged motifs have been identified in many of these proteins, including polarity regulators. These motifs are mostly composed of a stretch of positively charged Lysines and Arginines in the primary sequence but might also result from a three-dimensional clustering of more distant located amino acids upon protein folding. Due to their positive charge, these motifs interact electrostatically with the negatively charged phospholipids of the inner leaflet of the plasma membrane (reviewed in Li et al., 2014). Phenylalanine, Tryptophan and Leucin adjacent to positively charged amino acids further enhance the association with phospholipids (Heo et al., 2006). In contrast to the above-mentioned distinct lipid-binding domains, the affinity of polybasic motifs to particular phospholipids is diverse and (currently) hard (if not impossible) to predict if no three-dimensional structure of the protein is available. Calculation of a lipid-binding index of the primary protein sequence of a candidate protein might help to identify potential membrane binding sites (Brzeska et al., 2010), which subsequently needs be tested experimentally. However, three-dimensional polybasic motifs are not revealed by these predictions, which are based on the primary sequence. Due to structural differences of the head group of the phospholipids (Figure 2), the three-dimensional protein structure surrounding the polybasic motif might distinguish between PS, PA and the family of phosphoinositides. For the later, taking in account the charge density/concentration as well as its abundance in the plasma membrane (Balla, 2013), PI(4)P and PI(4,5)P2 are obviously the most targeted phosphoinositides (Gambhir et al., 2004). However, even short polybasic motifs might be as specific for one particular phospholipid as a specific lipid binding domain, e.g., the binding of the polybasic tail of LKB1 to PA is magnitudes higher than to PI(4,5)P2 and PI(3,4,5)P3 (Dogliotti et al., 2017).

## Apical Versus Basolateral Polarity: the Par-Complex and Its Antagonists

The scaffolding protein PAR-3 is essential for the initial establishment of apical-basal polarity by targeting PAR-6 and aPKC to the apical junctions. PAR-3 itself can be recruited to the adherens- and tight junctions by Cadherins (Harris and Peifer, 2005; Iden et al., 2006; Kullmann and Krahn, 2018b), Nectins (or the Nectin-like protein Echinoid in *Drosophila*) (Takekuni et al., 2003; Wei et al., 2005; Kullmann and Krahn, 2018b) and Junctional Adhesion molecule (JAM-A) (Ebnet et al., 2001). However, the correct subcellular localization of PAR-3 can also be accomplished by a conserved polybasic motif within the C-terminal part of the protein (Krahn et al., 2010b; Horikoshi et al., 2011; Ahmed and Macara, 2017; Kullmann and Krahn, 2018b). This Lysine/Arginine rich “lipid binding” motif binds to PI(4)P, PI(4,5)P2, and PI(3,4,5)P3 with highest affinity to the triphosphorylated PI. Deletion or mutation of this motif alone does not affect membrane localization of the full length protein, as the N-terminal oligomerization domain functions in redundancy to the lipid-binding motif in targeting PAR-3 to the apical junctions in *Drosophila* epithelial cells and neuroblasts (Kullmann and Krahn, 2018b). However, disturbed oligomerization together with impaired lipid-binding abolishes PAR-3 membrane association, which can be rescued by either PI(4,5)P2- or PI(3,4,5)P3-specific PH domains (Krahn et al., 2010b; Kullmann and Krahn, 2018b). Notably, plasma membrane association of PAR-3 is essential to prevent its proteasomal degradation (Kullmann and Krahn, 2018b). In *Drosophila* follicular epithelial cells, PI(4,5)P2 and not PI(3,4,5)P3 seems to be the phospholipid for correct apical PAR-3 localization, as depletion of the PI(4,5)P2-producing enzyme results in a mislocalization of PAR-3 to the basolateral membrane (but still remains membrane-associated) (Claret et al., 2014). Although the PDZ-domains of PAR-3 seem to have some phospholipid binding affinity (Wu et al., 2007; Yu and Harris, 2012), deletion of these domains do not affect membrane targeting in *Drosophila* epithelia *in vivo* (Krahn et al., 2010b; McKinley et al., 2012; Kullmann and Krahn, 2018b).

In epithelia and neuroblasts, PAR-3 is displaced from the basolateral membrane upon phosphorylation by laterally localized PAR-1 at two conserved Serines, one of which is located near the lipid binding motif (Benton and St Johnston, 2003). Subsequently to PAR-1 mediated phosphorylation, 14-3-3 adaptor proteins bind to phosphorylated PAR-3, which presumably blocks the lipid-binding domain, resulting in a membrane displacement of PAR-3 at PAR-1 active sites (Benton and St Johnston, 2003; Hurd et al., 2003a; Krahn et al., 2009).

Similar to the reciprocal regulation of PAR-1 versus PAR-3/aPKC, Lgl is phosphorylated by aPKC, which results in a displacement of Lgl from the cortex in epithelia and *Drosophila* neural stem cells (Betschinger et al., 2003; Plant et al., 2003; Wirtz-Peitz et al., 2008). Strikingly, the highly conserved aPKC-phosphorylation domain of Lgl was recently identified as a polybasic motif, which targets the protein to the plasma membrane by electrostatic interactions with phospholipids, in particular PI(4,5)P2 and PI(4)P (Dong et al., 2015). Phosphorylation of Lgl by aPKC attenuates the positive charge of this domain, thus inhibiting its interaction with phospholipids and displacing Lgl from the membrane (Bailey and Prehoda, 2015; Dong et al., 2015). In a similar mechanism, aPKC displaces Miranda and Numb from the plasma membrane (Bailey and Prehoda, 2015), which is essential for asymmetric cell division of neuroblasts. Both proteins contain polybasic motifs, too, with at least one internal aPKC-phosphorylation site, which blocks the interaction with phospholipids once it is phosphorylated. However, several other aPKC-phosphorylation sites have been identified in Miranda and Numb *in vitro* (Smith et al., 2007; Atwood and Prehoda, 2009). Although they are not situated in the polybasic motif, these sites partly contribute to aPKC-mediated membrane displacement of the proteins. This observation suggests either a stepwise phosphorylation, in which the critical polybasic motif phosphorylation site is only phosphorylated after a conformational change upon phosphorylation of the other critical sites. Or phosphorylation of residues outside the polybasic motif result in changes in protein folding so that the polybasic motif is masked by other domains of the protein itself or any of its interaction partners (which might be bound upon this particular phosphorylation event too). Three-dimensional modeling is needed to further understand the molecular mechanisms underlying these protein-membrane interactions. Notably, hypoxia reduces phosphoinositides in the inner leaflet of the PM, resulting in a decreased association of Lgl and Numb with the plasma membrane (Dong et al., 2015). This seems to be even more intriguing, as hypoxia is a key feature of rapidly proliferating tumor cells, which eventually lose their apical-basal polarity and cell-cell adhesion to become migratory.

In epithelial cells, not only proteins are asymmetrically distributed: PAR-3 at the apical junctions recruits the phosphatase PTEN, which converts PI(3,4,5)P3 to PI(4,5)P2 (von Stein et al., 2005; Feng et al., 2008), thereby supporting the segregation of apical enrichment of PI(4,5)P2 and basolateral PI(3,4,5)P3 in the plasma membrane (Martin-Belmonte et al., 2007). Strikingly, these PIs themselves function as polarity regulators: PI(4,5)P2 recruits Annexin-2 to the apical membrane, which assembles and activates Cdc42 and aPKC (Martin-Belmonte et al., 2007). Addition of PI(4,5)P2 to the basolateral domain in a three-dimensional cell culture model mistargets apical junctional markers to the basolateral membrane and vice versa: The addition of PI(3,4,5)P3 to the apical membrane domain recruits basolateral polarity markers to the ectopic PI(3,4,5)P3 microdomains. Conversely, inhibition of PTEN or PI3K results in polarity defects and disturbed lumen formation in three-dimensional cell culture models. In *Drosophila* tracheae and MDCK cysts, apical enriched PI(4,5)P2 together with RhoA recruits the Formin Diaphanous, which induces the formation of Actin cables necessary for apical secretion and thus tubulogenesis/lumen formation (Rousso et al., 2013). Furthermore, the authors showed that the PI(4,5)P2 producing PIP5K Skittles (in *Drosophila*) is apically localized and contributes to this process. These results indicate that PI(4,5)P2 and PI(3,4,5)P3 function as regulators of apical-basal polarity by specifically recruiting apical or basolateral polarity determinants.

Moreover, the asymmetric enrichment of phospholipids seems to be one of the first cues during the establishment of polarity: Peng et al. (2015) recently demonstrated that during polarization and lumen formation of MDCK cysts in a three-dimensional ECM matrix, PI3K (in particular, the p110δ isoform) is stabilized by Dystroglycan (DG) at the basal cell-matrix contact. Expression of p110δ is induced by ECM components and production of PI(3,4,5)P3 by p110δ is essential for the apical-basal polarization of the cell. Consequently, inhibition of p110δ with specific inhibitors results in an inverted polarity of the epithelial cells. Thus, phospholipid polarity and protein polarity are highly interconnected and regulate each other in order to accomplish apical-basal polarization of epithelial cells. Notably, this interaction between polarity proteins and phospholipids is also essential during oriented cell division: The junctional adhesion molecule JAM-A mediates the generation of PI(3,4,5)P3 by PI3K via an Cdc42-dependent pathway thus controlling the planar positioning of the mitotic spindle, which is tethered to the PI(3,4,5)P3-enriched cell poles by dynactin (Tuncay et al., 2015). Similar to protein polarity regulators, phospholipid polarity seems to be conserved throughout evolution and different forms of polarity: In anterior-posterior polarized *C. elegans* zygotes, PI(4,5)P2 accumulates asymmetrically at the anterior cortex, where it regulates actin assembly and the asymmetric localization of the anterior polarity proteins PAR-3, PAR-6 and PKC3 (aPKC) (Scholze et al., 2018). Conversely, downregulation of PAR-3, but not of the posterior determinant PAR-2, results in disturbed anterior PI(4,5)P2 accumulation.

## Cdc42 Is Recruited to the Plasma Membrane by Phospholipids for Full Activation

Cdc42 regulates a variety of cellular functions, including Actin dynamics and endocytosis, by which Cdc42 controls apical-basal polarity as well as cell migration. Notably, Cdc42 is one of the “oldest” polarity regulators in evolution as direct homologs can not only be identified in humans, flies and worms, but also in yeast.

Apart from its activation via membrane-bound Annexin-2, Cdc42 itself binds directly to PS via a polybasic motif at its C-terminus, and PS controls its asymmetric localization in budding yeast (Fairn et al., 2011; Das et al., 2012; Haupt and Minc, 2017). Furthermore, PS and GTPase activation of Cdc42 are essential for nano-clustering of the protein in budding yeast (Sartorel et al., 2018). Given the importance of Cdc42 in cell polarity it is likely that this interaction with phospholipids is also essential for apical-basal polarity of epithelial cells. However, it is still unclear whether or how PS-enriched microdomains within distinct plasma membrane domains contribute to asymmetric localization of proteins in epithelial cells.

## The Crb-Complex and Phospholipids

None of the two essential adaptor proteins of the Crb complex (Pals1 and PATJ) have been linked to phospholipids. However, Crb regulates phospholipid polarity by antagonizing the activity of PI3K in *Drosophila* epithelial cells, thus preventing apical conversion of PI(4,5)P2 to PI(3,4,5)P3 (Chartier et al., 2011). Interestingly, this seems to be one of the major functions of Crb as the polarity defects of *crb*-mutant embryos can be rescued to some extent by overexpression of PTEN or inhibition of PI3K. This fits with the hypothesis that Crb determines the apical [PI(4,5)P2-enriched] plasma membrane domain whereas PI(3,4,5)P3 in a positive feedback activation loop with Rac1 supports lateral membrane growth. Reciprocal activation of Rac1 and PI(3,4,5)P3 and their role in lateral membrane determination has also been shown for mammalian epithelial cells (Gassama-Diagne et al., 2006; Liu K.D. et al., 2007; Jeanes et al., 2009).

Apart from the canonical members of the Crb complex, several proteins have been linked to this complex biochemically. One important regulator of the complex is Angiomotin, whose expression is low in differentiated epithelia but becomes upregulated in migratory epithelial cells. Angiomotin binds to a PDZ domain of PATJ, disturbing its interaction with Crb-Pals1 (Wells et al., 2006). Notably, this process seems to be regulated by binding of Angiomotin to several phospholipids, with the highest affinity to PI(4)P, thereby targeting the Angiomotin/PATJ complex to recycling endosomes and thus disrupting the TJ, a prerequisite for cell migration (Heller et al., 2010). Therefore, phospholipid-binding of Angiomotin is a key regulatory mechanism of TJ/polarity disassembly during epithelial dedifferentiation (e.g., EMT), qualifying Angiomotin as an oncogene in some cancer types (reviewed by Lv et al., 2017).

## Posttranslational Modifications Regulate Plasma Membrane Localization of Lateral Polarity Regulators

Apart from the classical polarity complex Scribble/Dlg/Lgl, two conserved protein kinases localize to the lateral plasma membrane: PAR-1 and LKB1 (PAR-4 in *C. elegans*). Whereas PAR-1 antagonizes the PAR/aPKC complex (see above) and regulates microtubule dynamics (summarized by McDonald, 2014), LKB1 functions as a master kinase, phosphorylating the T-loop of several kinases of the AMPK (AMP-dependent Kinase) family, including PAR-1 (Lizcano et al., 2004). Consequently, phosphorylation of these kinases by LKB1 results in their full activation.

PAR-1 contains a C-terminal Kinase associated-1 (KA1) domain, which regulates the kinase activity of PAR-1 by auto-inhibition (Elbert et al., 2005). However, in 2006 the KA1 domain had already been identified as the region responsible for membrane association of the protein (Goransson et al., 2006). Later it became clear that this domain facilitates direct binding to PI(4)P, PS and PA (Moravcevic et al., 2010). Similar to polybasic motifs, positively charged Lysines/Arginines located at the outside of the folded KA1 domain are responsible for binding to negatively charged phospholipids. Mutation of these residues abolishes membrane association of PAR-1 in cell culture (Moravcevic et al., 2010). In the zygote of *C. elegans*, mutation of these residues displaces PAR-1 from the cortex (Ramanujam et al., 2018). Interestingly, similar to Lgl, phosphorylation of PAR-1 by PKC-3 (in worms) or aPKC (in *Drosophila* and mammals) results in a dissociation of PAR-1 from the membrane, providing a molecular mechanism for the aPKC < > PAR-1 exclusion (Hurov et al., 2004; Suzuki et al., 2004; Ramanujam et al., 2018). However, the aPKC-phosphorylation site is not located in the phospholipid-binding KA1-domain, suggesting an indirect effect of aPKC-mediated phosphorylation as discussed above for Miranda and Numb. Moreover, PAR-1 is phosphorylated outside the KA1 domain by several other kinases, and 14-3-3 adaptor proteins bind to phosphorylated PAR-1, displacing it from the membrane (Benton et al., 2002; Goransson et al., 2006). Consequently, the mutation of all phosphorylation sites results in a strictly membrane-association of PAR-1 (Goransson et al., 2006). Given the broad phospholipid binding of PAR-1 (Moravcevic et al., 2010), specific localization of PAR-1 might be rather accomplished by unspecific binding to the entire plasma membrane followed by apical exclusion (via the PAR/aPKC complex) than by selective targeting to the lateral plasma membrane domain.

LKB1 regulates an immense number of cellular processes, including energy metabolisms (via phosphorylating AMPK) and apical-basal/anterior-posterior polarity (by activating PAR-1 and AMPK). LKB1 has long been assumed to be a constitutively active kinase once it is associated with its co-factors Mo25 and SRADα. However, over the last years, several posttranslational modifications have been described (reviewed in Kullmann and Krahn, 2018a). Apart from a C-terminal farnesylation, which facilitates only transient membrane association, LKB1 contains a C-terminal polybasic motif adjacent to the farnesylated Cysteine, which directly binds to PA with a high specificity (Dogliotti et al., 2017). Mutation of this motif abolishes membrane association and severely decreases the kinase activity of LKB1 and the addition of PA-enriched micelles to recombinant LKB1 kinase complex increases its activity *in vitro*, and overexpression of Phospholipase D, one of the enzymes producing PA, results in an enhanced LKB1 activity in cell culture (Dogliotti et al., 2017). Notably, PDZGEF, a guanine exchange factor for Rap, also binds to PA (and other phospholipids) via a polybasic motif (Consonni et al., 2014). PDZGEF functions downstream of LKB1 to polarize intestinal epithelial cells by activating Ezrin via Rap2A (Gloerich et al., 2012). This suggests that PA-enriched microdomains in the PM function as signaling hubs, assembling several components of a distinct polarization pathway. However, it still remains unclear whether PA is enriched in distinct (micro)domains of the plasma membrane in apical-basal polarized epithelial cells. A possible hint comes from migrating epithelial cells, in which PA is enriched at the rear rather than at the leading edge (Ferraz-Nogueira et al., 2014), which would be in contradiction to the proposed role of LKB1 at the leading edge of migrating cancer cells (Zhang S. et al., 2008).

## Phospholipids and Polarity Regulators in Migrating Cells

In migrating (epithelial) cells, cell-cell adhesion molecules are downregulated or internalized and apical-basal polarity is converted into a front-rear polarity (Figure 1B). Now, the former apical PAR/aPKC complex accumulates at the leading edge (at least in isolated cells), being essential for collective cell migration as well as migration of fibroblasts (Etienne-Manneville, 2008; Schmoranzer et al., 2009; Mayor and Etienne-Manneville, 2016). However, in contrast to the apical domain of epithelial cells, the leading edge in migrating cells is characterized by accumulation of PI(3,4,5)P3, and conversion of PI(4,5)P2 to PI(3,4,5)P3 at the leading edge triggers the activation of Actin regulators in order to organize lamellipodia for forward movement of the cell. Like the majority of PM-localized small GTPases, the Actin cytoskeleton regulators Rho, Rac, and Cdc42 have been found to contain polybasic motifs by which they bind to PI(4,5)P2 and PI(3,4,5)P3 (Heo et al., 2006). The interplay and feedback loops between these phospholipids and Actin regulators is essential for cell migration (Maree et al., 2012). In order to prevent hydrolysis of PI(3,4,5)P3 to PI(4,5)P2, PI(3,4,5)P3 at the leading edge and PTEN at the rear edge are mutually exclusive, at least in *Dictyostelium discoideum* (Matsuoka and Ueda, 2018). Here, targeting of PTEN to the plasma membrane via interaction of its N-terminus with PI(4,5)P2 is essential for chemotaxis (Iijima et al., 2004), although PTEN also exhibits a C2 domain, which associates with PC and PS-enriched liposomes *in vitro* (Lee et al., 1999). This is in contrast to the situation in epithelial cells, where PTEN colocalizes and associates with apical-junctional PAR-3, which is found at the leading edge in migrating cells, far away from PTEN. One explanation could be that in epithelial cells, the border between PI(4,5)P2-enriched apical domain and PI(3,4,5)P3-accumulation at the basolateral domain might be below the resolution limit for current studies – in migrating cells, both poles (and thus PTEN and PAR-3) are spatially clearly separated.

Apart from its implication in Cdc42/Rac1-dependent Actin turnover, the PAR-complex also regulates microtubule dynamics and centrosome positioning in migrating cells via PAR-3/Dynein and Cdc42/PAR-6-activated aPKC (Etienne-Manneville, 2008; Schmoranzer et al., 2009). Disturbed aPKC activity does not affect front-rear polarity, but rather results in a random migration of the cell, whereas knockdown of PAR-3 leads to abolished cell migration in fibroblasts. However, it is still unclear whether PI(4,5)P2 or PI(3,4,5)P3-binding of PAR-3 is essential for its localization at the leading edge and its role during cell migration.

At the leading edge, Cdc42 and Rac1 regulate the polymerization, turnover and branching of Actin, thereby enhancing the formation of lamellipodia and filopodia (summarized by Sit and Manser, 2011). Mechanistically, the binding of the polybasic tail of Rac1 to PA, PI(4,5)P2 and PI(3,4,5)P3 results in the establishment of Rac1-nanoclusters, which are essential for the formation of lamellipodia (Remorino et al., 2017; Maxwell et al., 2018). One of the Rac1 activators, the GEF Tiam1, is recruited to the plasma membrane by PAR-3 in polarized and migrating epithelial cells (Chen and Macara, 2005; Pegtel et al., 2007). Interestingly, Tiam1 itself contains two PH-domains, which are capable of binding to PI(3,4)P2, PI(4,5)P2, and PI(3,4,5)P3 and thus tether Tiam1 to the plasma membrane, which is essential for its Rac1 activating function (Michiels et al., 1997; Ceccarelli et al., 2007). In contrast to Rac/Cdc42, Rho is active at the rear edge, supporting the establishment of focal adhesions and Actin stress fibers as well as inducing cell contractility by activating Myosin. Apart from their direct binding to phospholipids, numerous activating [Guanine Nucleotide Exchange factors (GEFs)] and inactivating [GTPase activating proteins (GAPs)] factors for small GTPases of the Rho family contain a BAR domain, which is activated by PM curvatures (reviewed in de Kreuk and Hordijk, 2012).

Similar to the PAR/aPKC-complex, the lateral polarity regulators Scribble/Dlg/Lgl and LKB1/PAR-1 are also essential for directed cell migration (reviewed by McDonald, 2014; Stephens et al., 2018). Like for PAR-3, it is not yet clear whether phospholipid-binding of the lateral polarity proteins (Lgl, PAR-1, LKB1) affects their function in this process. Notably, these “lateral” polarity complexes also accumulate at the leading edge, thus overlapping with the “apical” complex PAR/aPKC (at least in collective cell migration). This raises the question whether and why the mutual exclusion of aPKC – Lgl and aPKC/PAR-3 – PAR-1 described above does not occur at this subcellular compartment or whether the leading edge is further composed of microdomains with distinct polarity protein composition (and particular phospholipid enrichment).

## PI(4,5)P2 Targets Polarized Vesicle Trafficking

In migrating cells not only the polarization of Actin/Myosin dynamics is regulated by phospholipids of the plasma membrane – Thapa et al. (2012) described PI(4,5)P2 to be essential for the delivery of Integrins to the leading edge, where they are essential for the establishment of nascent focal adhesions, facilitating extension of the lamellipodium. Notably, the authors identified an interaction of the enzyme responsible for PI(4,5)P2 production, PI(4)P-5-Kinase type 1C, with the Integrin adaptor protein Talin to be essential for this process. Thus, although PI(3,4,5)P3 has been supposed to be the critical leading-edge PI, spatial and temporally regulated production of PI(4,5)P2 at the leading edge is essential to mediate the delivery of vesicles to sites of newly formed cell-matrix contacts. This vesicle delivery is accomplished by the exocyst complex, which is targeted to the plasma membrane by direct binding of its components Exo70 and Sec3 to PI(4,5)P2 (He et al., 2007; Liu J. et al., 2007; Moore et al., 2007; Zhang X. et al., 2008). Polarized vesicle delivery by the exocyst complex is also essential for apical-basal polarity (reviewed by Polgar and Fogelgren, 2018).

Interestingly, the polarity protein PAR-3 functions as an exocyst receptor (via SEC8) at the TJ, and the exocyst binding domain was mapped to the polybasic motif of PAR-3, which also mediates binding to phospholipids (Ahmed and Macara, 2017). Disruption of the PAR-3-exocyst interaction disturbs delivery of lateral transmembrane proteins (e.g., *E*-cadherin) und thus strongly affects apical-basal polarity. One interesting question would be whether PAR-3 can simultaneously bind to both, phospholipids and exocyst, or whether these bindings are mutually exclusive. Nevertheless, this study identified PAR-3 as a key gatekeeper for vesicle docking at the border of apical versus basolateral delivery, similar to its role in recruiting PTEN in order to separate PI(3,4,5)P3 and PI(4,5)P2-enriched membrane domains (see above). In the future, it would be interesting to test whether PAR-3 at the leading edge also functions as an exocyst receptor for the delivery of focal-adhesion proteins in migrating cells.

## Concluding Remarks

Emerging evidences suggest a critical role of phospholipids in regulating polarity proteins’ localization and function in polarized epithelial and migrating (epithelial and non-epithelial) cells and vice versa: Polarity proteins regulate the accumulation of distinct phospholipids in different membrane compartments by localizing the respective enzymes, e.g., PI3K accumulation at focal adhesions or PTEN targeting at apical junctions (von Stein et al., 2005; Martin-Belmonte et al., 2007; Feng et al., 2008; Peng et al., 2015). Thereby, the apical-basal polarity of phospholipids [PI(4,5)P2 apical, PI(3,4,5)P3 basolateral] is established and regulated. In contrast, there are increasing reports of phospholipids regulating the localization and function of polarity regulators suggest that phospholipid polarity is not only a simple consequence of protein polarity but both modules are affecting each other: Disturbed or manipulated phospholipid polarity results in mislocalization of proteins and impaired apical-basal polarity (e.g., Gassama-Diagne et al., 2006; Martin-Belmonte et al., 2007; Claret et al., 2014) and deletion/mutation of phospholipid-binding motifs in polarity regulators leads to mislocalization of these proteins and disturbed polarity (Moravcevic et al., 2010; Bailey and Prehoda, 2015; Dong et al., 2015; Dogliotti et al., 2017; Kullmann and Krahn, 2018b). Taken together, both mechanisms, phospholipid-regulated protein localization and – function and protein-directed phospholipids accumulation, are essential for cell polarization and probably affect each other reciprocally so that it is rather a positive feedback circuit than a simple cause-consequence relationship.

One complication in our understanding of these processes is that most polarity regulators bind to several phospholipids although with different affinities, which mostly have been tested *in vitro*. In many cases, it is still not fully understood which particular phospholipid(s) is/are essential for targeting of the polarity regulator to the plasma membrane. Furthermore, several phospholipids, in particular PA, PS, and PI(4)P have not been shown to be asymmetrically enriched in distinct (micro)domains of the plasma membrane in polarized epithelial cells. Nonetheless, their interaction with polarity regulators is essential for polarized localization of the proteins. One explanation is that other factors, such as displacement from the plasma membrane at other domains (as shown for Lgl, Numb, PAR-1 and Miranda) or binding of interaction partners masking the lipid-binding motif (as supposed for PAR-3), contribute to the correct targeting of polarity regulators.

Another obstacle is the current technical limitations: Some phospholipid-probes are not absolutely specific for one distinct phospholipid but also bind to several others with lower affinities, which might be enough to mask microdomains in the plasma membrane, in particular if the concentration of the phospholipid of interest is magnitudes lower than others. Secondly, the principle of probes itself is critical because (over)expression of these lipid-binding domains themselves may disturb the phospholipid composition of membranes and block targeting of polarity regulators. Enhanced sensitivity of microscopy techniques combined with super resolution/single molecule imaging and improved fluorochromes might diminish this problem in the future.

## Author Contributions

MK conceived and wrote the manuscript.

## Conflict of Interest

The author declares that the research was conducted in the absence of any commercial or financial relationships that could be construed as a potential conflict of interest.
